# Biology and Behavior of *Spathius agrili*, a Parasitoid of the Emerald Ash Borer, *Agrilus planipennis*, in China

**DOI:** 10.1673/031.010.3001

**Published:** 2010-04-06

**Authors:** Zhong-Qi Yang, Xiao-Yi Wang, Juli R. Gould, Richard C. Reardon, Yi-Nan Zhang, Gui-Jun Liu, En-Shan Liu

**Affiliations:** ^1^ The Key Laboratory of Forest Protection, State Forestry Administration of China, Research Institute of Forest Ecology, Environment and Protection, Chinese Academy of Forestry, Beijing 100091, China; ^2^ Animal and Plant Health Inspection Service, United States Department of Agriculture, 1398 West Truck Road, Buzzards Bay, MA 02542, USA; ^3^ Forest Health Technology Enterprise Team, USDA Forest Service, 180 Canfield St., Morgantown, WV 26505, USA; ^4^ Guangang Forest Park, Dagang District, Tianjin 300270, China

**Keywords:** Braconidae, *Fraxinus*, life cycle

## Abstract

*Spathius agrili* Yang (Hymenoptera: Braconidae) is a gregarious larval ectoparasitoid of the emerald ash borer, *Agrilus planipennis* Fairmaire (Coleoptera: Buprestidae) and is a recently described species. Both pest and parasitoid are native to China. In Tianjin City, China, *S. agrili* typically exhibited 3–4 generations per year, overwintering as a prepupa in a cocoon inside the host gallery. The multiple generations of *S. agrili* overlapped with its host, as did the emergence dates of the overwintering generation. From a single host, 1–18 *S. agrili* successfully developed to the adult stage (average 8.4), but in all cases the host was killed. The sex ratio (female: male) of the parasitoid adults emerging from field-collected cocoons was 2:1, whereas the sex ratio of parasitoids reared from field collected eggs and larvae was greater than 3:1. On average, adult females lived 29.1 d, and males lived 23.6 d when fed with 20% honey solution, significantly longer than without a nutritional supplement. Sexual reproduction is the normal mode of reproduction, but in the laboratory females did reproduce parthenogenetically, producing only males. The average fecundity was 23.3 eggs per female in the laboratory. *S. agrili* developed through five larval instars, and the larvae fed gregariously on the host hemolymph. The generation time from egg to adult wasp was 27–28 d at 22–26°C. Natural parasitism rates were as high as 60%, and in October they reached over 90% in some stands. This study showed that *S. agrili* is a promising agent for biocontrol of *A. planipennis.*

## Introduction

The emerald ash borer, *Agrilus planipennis* Fairmaire (Coleoptera: Buprestidae), is an important pest of the North American species of *Fraxinus* in China (Liu 1966, Yu 1992), and after its accidental introduction to North America it is a serious pest there as well (Haack et al. 2002). In China, the emerald ash borer has been reported in the northeastern provinces of Liaoning, Jilin and Heilongjiang, as well as in Shandong and Hebei Provinces and Tianjin Municipality. Damage was especially severe from 1999–2005 on velvet ash, *Fraxinus velutina* Torrey (Scrophulariales: Oleaceae), in tree plantations and along roadsides in Tianjin. Coastal barriers in many areas have plantings of this ash species that have been nearly eliminated by *A. planipennis* (Zhang et al. 1995, Liu et al. 1996). The concealed feeding and damage by *A. planipennis* larvae in the cambium of host trees often results in infestations going unnoticed until large ash stands are in critical condition and the trees die.

Early surveys by the senior author led to the discovery of *Spathius agrili* Yang (Hymenoptera: Braconidae), a previously undescribed parasitoid species and a potentially effective natural enemy of *A. planipennis* (Yang et al. 2005). *S. agrili* is a gregarious idiobiont ectoparasitoid. Preliminary field observations found parasitism rates of 30–90%, indicating that *S. agrili* might be an obligatory natural enemy of *A. planipennis* larvae (Yang et al. 2005). Further testing confirmed that *S. agrili* prefers *A. planipennis* as a host (Yang et al. 2008). *S. agrili* may be a key, natural factor keeping *A. planipennis* under control naturally in Tianjin, and it has high potential as a biocontrol agent. Herein, the life history, basic ecology and behavior of *S. agrili* are described, as studied in its native range in Tianjin, China, during 2003 and 2004.

## Materials and Methods

### Insect collection and rearing

*A. planipennis* larvae were sampled from a 5 ha stand of 9- to 10-year-old *F. velutina* spaced 1.0 m apart in parallel rows with 1.5 m spacing in Guangang Forest Park (38°56′N, 117°29′E), Dagang District, Tianjin Municipality, China. In August 2003, a total of 12 *F. velutina* were debarked to locate and collect *A. plenipennis* larvae, which were then transported to the laboratory. Collected larvae were fed fresh ash twigs that were 1.0–1.5 cm in diameter and 5–10 cm in length. Each ash twig was split in half lengthwise with one-half having a 3 cm long groove excavated through to the bark. A field-collected *A. plenipennis* larva was placed in each groove, and the other half of the twig was replaced and secured with rubber bands. The two ends of each ash tree “rearing chamber” were sealed with wax to prevent desiccation of the plant tissue during feeding of *A. plenipennis* larva.

*S. agrili* cocoons were collected from their host galleries. Approximately 10 clutches were collected every 3 days from June 18 to August 28, 2003. Adult wasps that emerged from these cocoons were cultured in the laboratory and fed a 1:4 honey: water solution.

### Adult emergence

*S. agrili* cocoons from individual host galleries were collected on March 7, April 15, and May 7 2004 and placed into single test tube (1.5 × 10 cm), one clutch per tubes. A small piece of moistened filter paper was placed in each tube to maintain humidity.

Tubes were held in the laboratory at room temperature (average 22.3°C, max 26°C, min 18°C) and checked daily for adult emergence. Surveys of forest areas were made using a sweep net and visual observation of tree trunks every 3 d beginning in late May to establish the first appearance of adults.

### Adult life history

Adult longevity, sex ratios, mating behaviors, and phototaxis were determined from laboratory and field observations. Longevity of adults in the laboratory was determined using three treatments: 1) supplying 20% honey solution on pieces of cotton, 2) water only without honey, and 3) no nutrition or water provided. Mortality was recorded daily. No fewer than 14 adults of each sex were used per treatment.

Fecundity was measured by placing newly-emerged male and female pairs of adult *S. agrili*, along with an *A. planipennis* larva in an excavated ash twig, in glass test tubes (2.8 × 11.5 cm) sealed tightly with cotton plugs. *A. planipennis* larvae were examined every 2–3 d to determine if parasitoid eggs were laid. If *S. agrili* eggs were observed on an *A. planipennis* larva, they were counted, and an unparasitized host larva was provided in a new twig. Two treatments were used: adults supplied a 20% honey solution and adults provided water only. Pre-oviposition and oviposition periods and fecundity were recorded for each female wasp. The rate of oviposition was defined as the percentage of females in a given treatment group that laid eggs. A total of 40 pairs were observed; 17 pairs were provided the honey solution, and 23 pairs had access to water but no honey.

### Determination of larval instars and stadia

*S. agrili* eggs were separated from the clutch of eggs and placed individually on un-paralyzed *A. planipennis* larvae that were in excavated ash twigs. The *S. agrili* eggs were separated because it would have been difficult to observe egg hatch and molting of larvae developing communally. The eggs were checked three times per day at 8:00, 15:00, and 22:00 h. First-instar larvae were placed into glass test tubes (1.5 × 7 cm) and reared individually on un-paralyzed *A. planipennis* larvae. Molts were monitored by marking the exuviae of the parasitoid larvae with black oil paint and examining them three times per day under the “Motic” stereo microscope (Motic, www.motic.com). When an exuvia marked with black oil paint was shed, the molt was recorded and the fresh exuvia was marked for further observation. Body length and width were determined for each larval instar using an ocular micrometer under the “Motic” stereo microscope. Observations were made within 7 h after the insect molted and continued until pupation occurred. The duration of the pupal stage was also recorded. The development of twenty insects was recorded using the methods described above.

### Parthenogenesis

Newly emerged, virgin *S. agrili* females were placed individually in glass test tubes (2.8 × 11.5 cm) with their host larva in an excavated ash twig. The test tubes were sealed with cotton plugs, and host larvae were examined every 2–3 d for eggs. When *S. agrili* eggs were found on an *A. planipennis* larva, they were counted, and an unparasitized *A. planipennis* larva in a fresh twig was provided. Two treatments were used: adults supplied with 20% honey solution and adults provided water only. The number of eggs laid, offspring development (percentage survival from egg to pupa), and offspring sex were recorded. Each treatment was replicated using 10 female *S. agrili.*

### Life cycle and population dynamics in forests

In the research plot starting in late May, field surveys were conducted every 3 d until late October, when *A. planipennis* had become prepupae and entered their pupal chambers in the shallow sapwood for overwintering. During each survey, 5–10 ash trees were sampled at random for *A. planipennis* larvae and their parasitoids. A 10 × 30 cm bark window was removed from the trunk of each tree at 4–6 locations between 0.5–1.5 m. *S. agrili* eggs, larvae, and pupae associated with *A. planipennis* larvae or its gallery were counted and transported to the laboratory to rear. The parasitism rate (percentage of *A. planipennis* parasitized) and the number of *S. agrili* per *A. planipennis* larva were calculated for each survey. The life stage development and population dynamics of *S. agrili* in the forest were estimated from field observations combined with information on the duration of each stage observed in the laboratory.

### Dissection of ovarioles of adult female

One-week-old female *S. agrili* that emerged from cocoons collected in field were killed with ethyl acetate vapors after mating but prior to oviposition. Specimens were fixed on a wax dish (7.0 cm diameter), immersed in 1% NaCl solution, and dissected under the “Motic” stereo microscope (80X magnification). The structure of the female reproductive system and the number of eggs contained in the ovarioles were recorded.

### Data analysis

Data were analyzed with SAS version 9.1.3 (SAS Institute Inc. 2006) for variance analysis by PROC GLM program. The mean values of the wasp adult longevity, preoviposition period, oviposition period, egg production, etc., under different conditions were compared using analysis of variance, and a least significant difference multiple range test was run for separation of means. Sex ratio and the rate of oviposition (percentage of females that laid eggs) between different groups were compared using the chi square method.

## Results and Discussion

### General life history

**Figure 1.**
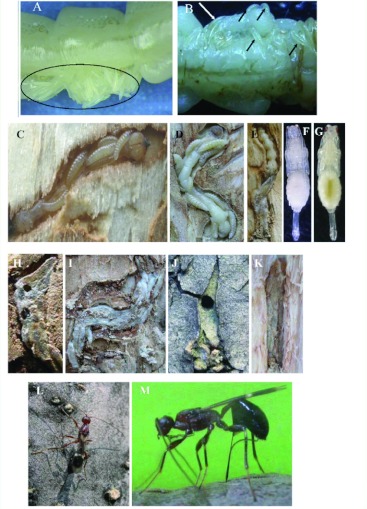
*Spathius agrili* life stages and behavior. A) eggs laid on the surface of *Agrilus planipennis* larva; B) eggs (white arrow) and newly hatched larvae (black arrows); C) larvae feeding; D) full-grown larvae and remains of their host; E) cocoons and remains of host larva; F) young pupa; G) mature pupa; H) cocoons with emergence holes; I) cocoons filling *A. planipennis* gallery; J) emergence hole in bark chewed by emerging parasitoid wasp; K) cocoons in host overwintering chamber in the shallow sapwood; L) adult female searching for hosts; M) female ovipositing. High quality figures are available online.

The life-cycle of *S. agrili* began in the summer with the emergence of adults. The female wasps searched for hosts by walking on the trunks and/or twigs of ash trees, tapping the bark with their antennae (Figure 1L). When a host was located, the female passed her ovipositor through the bark into the *A. planipennis* gallery (Figure 1M), paralyzed the *A. planipennis* larva by injecting venom, and then laid a clutch of 1–35 eggs on the host surface (Yang et al. 2005) (Figure 1A). After hatching, *S. agrili* larvae attached themselves to the paralyzed host larva, feeding externally (Figures 1B, 1C, 1D). Mature wasp larvae spun silken cocoons (Figures 1E, 1I) within which they pupated (Figure 1F, 1G), and adults chewed through the bark upon emergence (Figure 1H, 1J). *S. agrili* overwintered as prepupae inside cocoons in its host gallery. A brood of cocoons usually occupied the space previously occupied by the host larva, which was reduced to a thin thread after being fed upon by the parasitoid (Figure 1I).

### Development of life stages

*S. agrili* eggs were white, semitransparent, elongate, and slightly curved, with one end rounded and the other end narrowing (Figure 1A). Eggs were 0.8 ± 0.02 mm in length and 0.1 ± 0.002 mm in width (Table 1). The parasitoid eggs were typically laid together as a clutch on the body surface of *A. planipennis* larvae. A fluid was exuded by the ovipositing female onto the surface of the eggs for gluing and attaching the eggs to the host larvae. Most eggs hatched in 1–3 d (Figure 1B) with a mean of 1.6 ± 0.16 d at room temperature (22–26°C).

**Figure 2.**
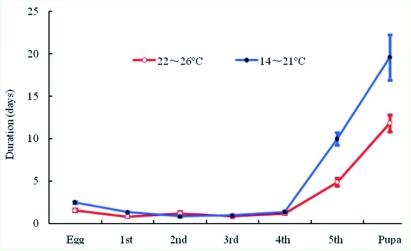
Developmental duration of *Spathius agrili* eggs, five larval stages, and pupa at two temperatures. High quality figures are available online.

Larvae were cream-colored and had 14 segments with spiracles that were not clearly visible (Figure 1C, 1D). There were five larval instars, and the duration of each instar is presented in Table 2. At lower temperatures 14–21°C, the duration of the fifth instar and pupa increased (Figure 2). Fifth instar larvae grew from an average of 4.4 mm (range 3.0–5.5 mm) shortly after the molt to 6.17 mm (range 5.0–7.0 mm) just prior to pupation. *S. agrili* larval feeding in the laboratory was impacted by the manipulation during monitoring and frequent marking. It was difficult for the 4th or 5th instar *S. agrili* larvae to feed on its un-paralyzed host after introduction into the test tube because the *A. planipennis* larvae resisted by twisting their body and crawling. Naturally occurring larvae did not encounter this problem because the hosts were paralyzed by the ovipositing female. The placement of early-instar larvae of *S. agrili* onto the body of *A. planipennis* larvae did not elicit avoidance behavior by the *A. planipennis* larva.

**Table 1.**
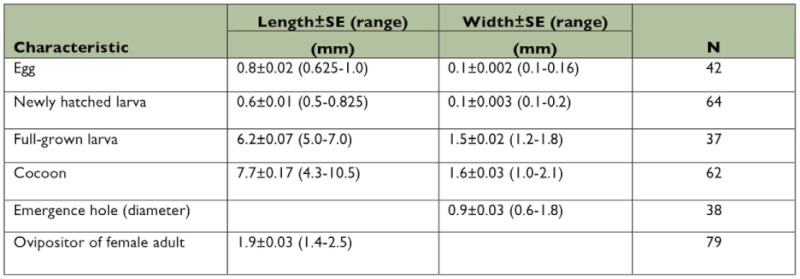
Measurements of *S. agrili* life stages, cocoons, emergence holes and female ovipositors for parasitoids collected in the field.

After feeding for 8–10 d, the parasitoid larvae became 5th instars, and during this stage they consumed virtually all of the host, leaving only its exuvia, which was shrunken and thread-like. The wasp larvae then aggregated, spun and formed adjacent cocoons inside the host gallery (Figure 1I), and pupated. The overall shape of the aggregative brood of cocoons assumed the form of the mature host larva at end of the gallery. In some cases the host larva was not completely consumed because there were only a few feeding *S. agrili* larvae (usually fewer than four individuals) (Figure 1E). Cocoons of the overwintering generation were typically brown and thicker than those of the non-overwintering generations, which were cream to light brown and thinner walled. The cocoons were 4.3–10.5 mm in length and 1.0–2.1 mm in width (Table 1). At room temperature (average 20.7° C, min 13.5° C, max 29.6°C), the duration of the pupal stage was 11–29 d with a mean of 17.4 ± 0.46 d (*n* = 83). Pupation was shorter at higher temperatures (11–13 d in August and 18–20 d in September).

The exarate pupae (Figure 1F) were cream colored. Prior to emergence, the compound eyes became red (Figure 1G), and then, beginning at the head, the body darkened until the abdomen and ovipositor appeared black. The ovipositor lay tightly against the abdomen until eclosion of the female *S. agrili.* Four to six d were required from cocoon formation to pupation in August under room conditions (average 26.8°C, min 23.1° C, max 29.8°C). When food was inadequate (i.e., host too small to support that number of parasitoids) pupation could be delayed and cocoons might not be formed prior to pupation.

**Table 2.**
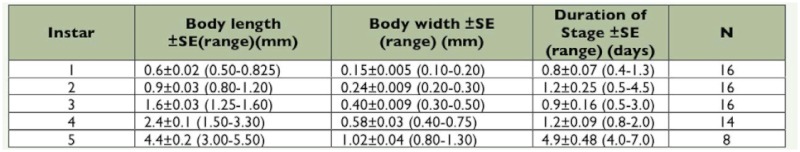
The body lengths, widths and durations of *S. agrili* larval stages reared at 22–26°C

**Table 3.**
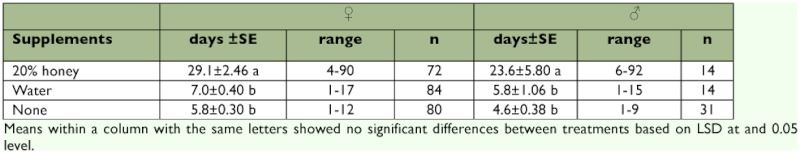
Longevity of *S. ogrili* adults at 21–30°C, provided 20% honey solution, water only, or neither

Adult *S. agrili* were 3–5 mm in length and black-brown, with yellow legs and antennae with 25 segments (Fig. 1L, 1M). Females were slightly larger than males, with a 2 mm long ovipositor. Adult *S. agrili* seldom moved at temperatures below 15°C. They were noticeably more active at temperatures above 18° C and appeared fully active at 25–30°C (i.e., actively crawling, flying, courting, and mating). The active adults were positively phototatic, which was more strongly exhibited by females than males.

At temperatures of 21–30° C (average 24.7°C), the lifespan of female and male *S. agrili* adults was an average of 29.1 ± 2.5 d and 23.6 ± 5.8 d, respectively, when fed a 20% honey solution (Table 3). This was significantly greater than for adults that had access to water only or that were reared without water or honey. Some individuals fed the honey solution lived as long as 90 d. Not surprisingly, adult longevity was longer when temperatures were lower. Female *S. agrili* usually lived longer than males under the same conditions (Wang et al. 2007). Virgin *S. agrili* females reproduced parthenogenetically, producing only male offspring. The oviposition rate for unmated females fed a honey-water solution was 60%, while it was 20%) for unmated females provided water only.

### Mating

Males of *S. agrili* typically eclosed prior to females, chewing an exit hole through the bark. In the same brood, males emerged prior to females 78.4% of the time. Males waited on the bark outside of the pupation chamber for mating opportunities with females exiting from the same hole. The emergence period for a single brood ranged from 5 to 44 d (*n* = 56) with a mean period of 18.2 ± 1.18 d. This lengthy period suggested that not all mating occurred between siblings.

Male courtship behavior was most frequent when room temperatures reached 26–30°C, with frequent crawling, jumping, wing beating, and pursuing of females. In copulation, males clasped the female's dorsum, beat their wings, and tapped females' antennae vigorously with their own antennae, while the females were inactive. Mating time ranged from 2 to 35 s with a mean of 16 ± 3.8 s. Some males were observed to mate with multiple females during the observation period, but females usually avoided mating again after they mated once. After mating, females usually moved away from courting males. Females that did mate multiple times were paired with aggressive males that pursued them. When several males and females were introduced into a single test tube, apparent competitive mating behavior among males was exhibited with males often attacking copulating pairs. During field observations in August between 8:00–11:00 and 15:00–18:00 h, female and male groups with upwards of 10 individuals were observed on single tree trunks. Mating and oviposition were observed during this period in the same areas on trees.

**Figure 3.**
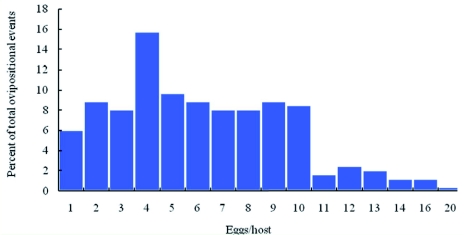
Percentage of various clutch sizes of *Spathius agrili.* High quality figures are available online.

### Oviposition

Following mating, the pre-oviposition period averaged 9.9 ± 2.9 d, with some females laying eggs on day three and other females laying their first egg after 28 days. The oviposition period typically lasted 8–18 d with a maximum of 47 d. Individual *S. agrili* oviposited up to eight times on eight hosts, depositing 1–10 eggs (average 5.8 ± 0.24 eggs/host, *n* = 193) in each clutch, although up to 20 eggs per clutch was observed (Figure 3). *S. agrili* has been shown to lay more eggs on larger *A. planipennis* larvae (Wang et al. 2008), sometimes even parasitizing mature *A. planipennis* larvae that were partially or completely inside of the overwintering chamber, which was in the shallow sapwood (Figure 1K). First and second instar host larvae were avoided, probably because small *A. planipennis* larvae would not provide sufficient nutritional resources for the development of multiple parasitoid larvae. Single parasitoid females oviposited a total of 6–68 eggs over their lifetime. Fecundity was higher for females that received honey solution supplements, but the differences were not significant. In the honey treatment there were 17 pairs of wasps, and 12 females laid eggs; while in the water treatment there were 23 pairs of wasps but 11 females laid eggs. The χ^2^ -test results comparing percentage of females that oviposited showed that there was no significant difference between treatments (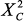 = 1.2457 < 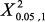 = 3.84) (Table 4). The ovarioles of a 1-week-old female usually contained only a few mature eggs (mean 8.5 ± 0.87 eggs, range 6–15 eggs, n = 12). Because females could lay eggs multiple times, and because the total eggs laid on hosts was much greater than the number of mature eggs observed at the onset of oviposition, *S. agrili* was determined to be synovigenic.

**Table 4.**
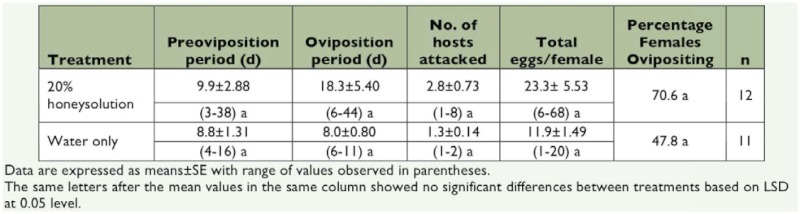
Oviposition by *S. agrili* females which were fed with 20% honey solution or water

Based on field and laboratory observations of eggs, larvae, and pupae, the clutch size of *S. agrili* was higher in forests than in the laboratory. In one instance, 35 eggs were found deposited on a mature field-collected host larva, although the number of ovipositional events that were involved could not be determined.

Female wasps were observed in the field tapping the bark with their antennae as they actively searched the trunks and twigs to locate host larvae for oviposition (Figure 1L). This activity typically peaked between 8:00–11:00 and 15:00–18:00 h, being greatest on sunny days when more than 10 individuals were regularly observed on a single trunk. After locating a host larva, the female inserted her ovipositor (length 1.4–2.5 mm, mean 1.9 ± 0.03 mm) completely through the bark (Figure 1M), piercing the host larva and paralyzing it with venom. The process required approximately 20 min. Eggs then were laid on the host's body surface (Figure 1A), during which another 40 to 90 min elapsed. During the egg-laying process, the female parasitoid was observed to contract rhythmically and shake her abdomen, which might aid in the movement of eggs through the egg channel in the ovipositor. The female remained at the site 10–20 min after removing her ovipositor from the bark. It is unlikely that the females deposited an oviposition deterrent pheromone because the paralyzed host larvae no longer feed, and *S. agrili* had been shown to oviposit only on larvae that are feeding (Wang et al., unpublished data).

**Figure 4.**
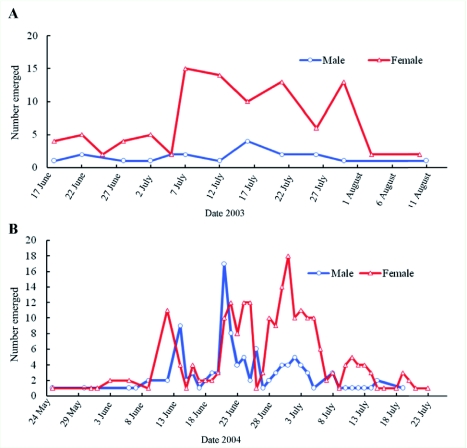
Emergence of overwintered *Spathius agrili* from cocoons collected from field plots in A) 2003 and B) 2004 and

### Field observations of adult emergence, brood size, sex ratio, and parasitism rates

Adult *S. agrili* chewed an exit hole of approximately 1 mm in diameter in their cocoon to escape (Figure 1H) when they emerged. After exiting the cocoons, *S. agrili* then bit a round hole through the bark (mean 0.9 ± 0.03 mm diameter, range 0.6–1.8 mm diameter) (Figure 1J) and emerged. Typically, only one exit hole was made per brood, although two or three were observed occasionally. Adults emerged most often at the 15:00 sampling period.

Adults of *S. agrili* first appeared in Tianjin ash forests in late May (2004) or June (2003) and continued emerging for over 2 months (Figure 4). In both years, emergence was low during the first 2–3 weeks, when *A. planipennis* larvae were early instars and unable to support feeding by the usually gregarious *S. agrili* larvae. Beginning in early July 2003 and early June 2004 there was a large increase in *S. agrili* emergence that lasted for approximately one month. It was during this period that the preferred older instars of *A. planipennis* became available. Studies have shown that *S. agrili* adults emerge up to several months after *A. planipennis* adults to allow for development of the preferred host stage, the late instar larvae (Wang et al. 2006).

**Table 5.**
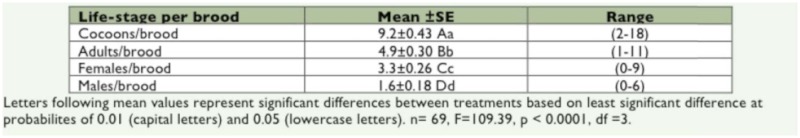
Number of *S. agrili* cocoons and adults from broods collected in the field

**Table 6.**
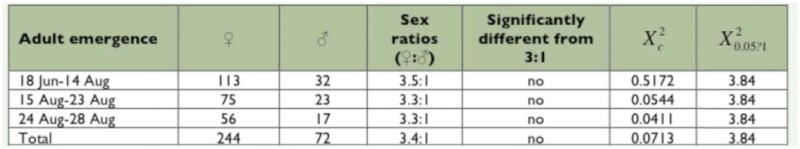
Sex ratios of *S. agrili* sampled August 2003 in Tianjin

**Figure 5.**
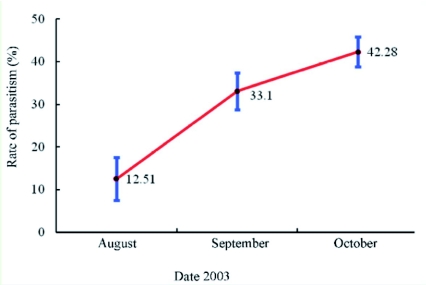
*Spathius agrili* parasitism rates in Tianjin represented as mean values from eight to ten surveys each month in 2003. High quality figures are available online.

**Figure 6.**
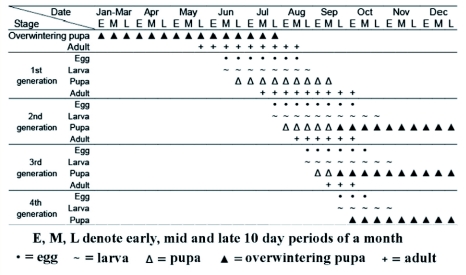
Life history of *Spathius agrili* in Tianjin, 2003–2004. High quality figures are available online.

Field-collected single broods of *S. agrili* consisted of 2–18 cocoons (average of 9.1 per brood). Sex ratios were highly variable during the emergence period within and between years (Figure 4). When data were pooled, females outnumbered males 2.1 to 1 (Table 5). Sex ratios were also determined from a total of 316 adults of *S. agrili* that were reared in the laboratory from field-collected eggs and larvae. Adult emergence was grouped into three consecutive periods during the summer of 2003, and sex ratios (female: male) ranged from 3.3:1 to 3.5:1 (Table 6). There was no significant difference among these sex ratios or individually from 3:1.

The parasitism rate of the first *S. agrili* generation was relatively low, about 10%. However, parasitism gradually increased and peaked with the overwintering generation in the late year, usually over 40% (Figure 5), with parasitism in some forest stands exceeding 90%. During 2003 in Tianjin, adult *S. agrili* were sampled by sweep net as late as October 10, but at that time they appeared much less vigorous and typically died soon in the laboratory. These adults may have been part of the last generation of the season.

### Number of generations per year

At ambient room temperature of 18–27° C (average 22.4°C), the development duration from egg to adult stages was 27–28 d, with the mean 27.9 ± 0.63 days (range 25–34, *n* = 26) for females and 28.2 ± 1.38 days (range 26–35, *n* = 6) for males. Combining estimations of developmental durations under laboratory conditions with field temperatures recorded during observation, the occurrence of all generations of *S. agrili* were estimated (Figure 6). It was predicted that *S. agrili* could complete 3–4 generations per year in the forests of Tianjin, China.
